# An LRP16-containing preassembly complex contributes to NF-κB activation induced by DNA double-strand breaks

**DOI:** 10.1093/nar/gkv161

**Published:** 2015-03-03

**Authors:** Zhiqiang Wu, Chunmeng Wang, Miaomiao Bai, Xiaolei Li, Qian Mei, Xiang Li, Yao Wang, Xiaobing Fu, Guangbin Luo, Weidong Han

**Affiliations:** 1 Department of Molecular Biology, Institute of Basic Medicine, Chinese PLA General Hospital, Beijing, 100853, China; 2 Department of Genetics & Genome Sciences, Case Comprehensive Cancer Center, University Hospitals of Cleveland and Case Western Reserve University, Cleveland, OH 44106, USA

## Abstract

The activation of NF-κB has emerged as an important mechanism for the modulation of the response to DNA double-strand breaks (DSBs). The concomitant SUMOylation and phosphorylation of IKKγ by PIASy and ATM, respectively, is a key event in this mechanism. However, the mechanism through which mammalian cells are able to accomplish these IKKγ modifications in a timely and lesion-specific manner remains unclear. In this study, we demonstrate that LRP16 constitutively interacts with PARP1 and IKKγ. This interaction is essential for efficient interactions among PARP1, IKKγ, and PIASy, the modifications of IKKγ, and the activation of NF-κB following DSB induction. The regulation of LRP16 in NF-κB activation is dependent on the DSB-specific sensors Ku70/Ku80. These data strongly suggest that LRP16, through its constitutive interactions with PARP1 and IKKγ, functions to facilitate the lesion-specific recruitment of PARP1 and IKKγ and, ultimately, the concomitant recruitment of PIASy to IKKγ in response to DSB damage. Therefore, the study has provided important new mechanistic insights concerning DSB-induced NF-κB activation.

## INTRODUCTION

DNA double-strand breaks (DSBs) can lead to genome instability and cell death. In mammals, genome instability can lead to oncogenic alterations, whereas excessive cell death can have serious consequences on the well-being of affected individuals. However, DSBs are also an intermediate in several important processes, such as V(D)J recombination, class-switch recombination and meiosis; and a byproduct of a number of both normal or pathological conditions, such as metabolic respiration and inflammation (1). Thus, the determination of the proper fates of cells with this specific type of DNA damage, i.e. to repair and survive or to die, constitutes an important element in the homeostasis of individual mammals.

The so-called DNA damage response (DDR) signal transduction cascade plays a critical role in determining the fate of cells with DNA damage (2). On the one hand, the activation of this cascade results in a series of events that play important roles in promoting the repair of DNA damage; whereas on the other hand, it also leads to the activation of either the pro- or anti-apoptotic pathways that ultimate determine the fate of the damaged cells. Several effectors, including Ataxia Telangiectasia Mutated (ATM), DNA-PKs and Poly (ADP-ribose) Polymerase 1 (PARP1), play pivotal roles in this signal transduction cascade (1). In particular, the activation of ATM plays a very important role in both the repair of DSB and the fate of the DSB-containing cells (3). ATM is a member of the PI3 family of protein kinases. Under normal conditions, ATM is accumulated in an inactive dimeric form primarily in the nucleus but also in the cytosol. In the presence of DSB, ATM is rapidly recruited to a DSB through its intrinsic affinity to DNA as well as through physical interaction with the MRN complex. The MRN complex consists of MRE11, Rad50 and NBS1 and has a high affinity for DSBs. The binding of ATM to the MRN complex results in its phosphorylation and the activation of the DDR signal transduction cascade (4).

The activation of the ATM-dependent DDR signal transduction cascade is well known for its effect on the activation of the CHK1 cell cycle checkpoint, a number of proteins that are involved in DSB repair, and the activation of the p53 transcription factor (5,6). P53 regulates a series of target genes that play important roles in cell cycle control, survival and apoptosis. Thus, historically, the ATM-p53 pathway is well known for its critical role in determining the fate of DSB-containing cells, although the mechanistic detail has not been fully understood. Interestingly, several recent studies have uncovered a new paradigm in which ATM affects cell survival in response to DSB induction, namely the so-called DNA damage-induced activation of NF-κB (7–9). In this unique paradigm, the DSB-dependent activation of ATM plays a critical role in the SUMOylation and phosphorylation of IKKγ within the nuclei. These modified IKKγ proteins are then exported to the cytosol. The presence of such modified IKKγ proteins is central to the activation of the NF-κB pathway as it is a requisite for the phosphorylation and activation of the IKKα/β kinase complex. In the classical paradigm of NF-κB activation, such IKKγ modifications occur in the cytoplasm following the binding of the appropriate ligands to specific cell surface receptors (10,11).

Intriguingly, this DNA damage-induced NF-κB activation paradigm also requires PARP1-mediated poly-ribosylation (8). Upon DSBs induction, PARP1 synthesizes poly (ADP-ribose) (PAR) from a donor nicotinamide adenine dinucleotide (NAD^+^) upon itself as well as other proteins. The PAR of the modified PARP1 then provides a scaffold for the assembly of the initial signalosome required for the simultaneous SUMOylation and phosphorylation of IKKγ to initiate the activation of the DNA damage-induced NF-κB signal transduction cascade (12). Remarkably, it has remained unclear how DSBs can lead to the actions of such a large number of factors in such a precise temporal and spacious coordination. In this study, we have demonstrated that Leukemia Related Protein 16 (LRP16) plays a unique and important role in facilitating this coordination.

LRP16 is a member of the macro domain family (13,14). Members of this family of proteins have been implicated in an array of diverse biological functions ranging from transcriptional regulation and chromatin remodeling to DNA damage repair and response (14–18). Our previous studies show that LRP16 is an essential cofactor of multiple nuclear receptors (19,20) and for the NF-κB transcription factor (21). In this manuscript, we report an important role of LRP16 in the regulation of DNA damage-induced NF-κB activation. We showed that LRP16 constitutively interacted with PARP1 and IKKγ. Treatment with DSB-inducing agents, such as etoposide (VP16), or exposure to ionizing radiation was found to result in significant enhancements of these interaction and activation of the NF-κB signal transduction cascade. Conversely, LRP16 silencing diminished such interactions and reduced the levels of NF-κB activation and cell survival following the induction of DSBs. Together, these data establish a critical role of LRP16 in the DSB-induced activation of the NF-κB signal transduction cascade to promote cell survival. Moreover, we found that LRP16 also interacted physically with Ku70 and Ku80, and that the depletion of Ku80 resulted in a significant reduction in the physical interactions among LRP16, PARP1 and IKKγ. Additionally, the knockdown of either endogenous Ku80 or Ku70 by siRNA markedly diminished DSB-induced NF-κB reporter gene activity and NF-κB target gene expression. Thus, our data also suggest that the Ku70/Ku80 complex plays a critical role in the activation of the NF-κB signal transduction cascade in response to DSB induction and that LRP16, through its unique physical interactions, plays a key role in orchestrating a series of events that ultimately lead to the concomitant recruitment of ATM and PIASy to IKKγ to ensure NF-κB activation following the induction of DSBs. Therefore, this study also provides important new mechanistic insights regarding the regulation of the DNA damage-induced NF-κB pathway.

## MATERIALS AND METHODS

### Reagents and plasmids

Etoposide, 3-aminobenzamide (3-AB), benzamide (BEN), PJ-34, ethidium bromide and human recombinant TNF-α were purchased from Sigma. Doxorubicin and camptothecin were obtained from KeyGEN Biotech. Lipofectamine 2000 and Superfect Transfection Reagent were obtained from Invitrogen and Qiagen, respectively. The Dual-Luciferase^®^ Reporter Assay Kit was obtained from Promega. Protein A agarose (16–156) was obtained from the Millipore Corporation. PhosSTOP Phosphatase Inhibitor Cocktail Tablets (04906845001) and Complete Protease Inhibitor Cocktail Tablets (4693116001) were obtained from Roche Applied Science. Biotin-NAD+ (4670–500–01), NAD (4684–096–02), human PARP enzyme (4668–100–01), activated DNA (4671–096–06) and PARP buffer (4671–096–02) were purchased from Trevigen. GST·Bind Resin (70541–3) was obtained from Novagen. CCK-8 was obtained from Dojindo. The Annexin V-FITC Apoptosis Detection Kit was obtained from BD Bioscience. The following antibodies were used in this study: sc-74470 (anti-PARP1, Santa Cruz Biotechnology); 9532 (anti-PARP1, Cell Signaling Technology); 2078 (anti-phospho-IKKα (Ser176)/IKKβ (Ser177), Cell Signaling Technology); ab63551 (anti-IKKγ (phospho S85), Abcam); sc-166744 (anti-PIASy, Santa Cruz Biotechnology); sc-5308 (anti-SUMO-1, Santa Cruz Biotechnology); sc-8330 (anti-IKKγ, Santa Cruz Biotechnology); sc-372 (anti-p65, Santa Cruz Biotechnology); sc-371 (anti-IκBα, Santa Cruz Biotechnology); 2370 (anti-IKKβ, Cell Signaling Technology); sc-5280 (anti-Ku80, Santa Cruz Biotechnology); sc-5309 (anti-Ku70, Santa Cruz Biotechnology); sc-1616 (anti-β-actin, Santa Cruz Biotechnology); sc-420 (anti-Sp1, Santa Cruz Biotechnology) and sc-138 (anti-GST, Santa Cruz Biotechnology); sc-56198 (anti-pADPr, Santa Cruz Biotechnology); Alexa-Fluor 488 goat anti-rabbit IgG and Alexa-Fluor 594 goat anti-mouse IgG (Life Technologies).

The wild-type LRP16-expressing construct has been described in our previous report (22). siRNA-resistant LRP16 was cloned through PCR amplifications into pCDNA3-FLAG. The dominant-negative mutant IκBm, in which serine 32 and 36 are mutated to alanine, was inserted into pCDNA3 with a flag tag. The luciferase reporter 3×κB-luc was a gift from Professor Chen Wang (Chinese Academy of Science) (23). The identities of all of the constructs were verified by sequencing analyses.

### Cell culture and transfection

HeLa cells and HT-29 cells were grown in RPMI 1640 and C33A cells and MCF-7 cells were grown in DMEM. Both were supplemented with 10% fetal calf serum and 100 U/ml penicillin/streptomycin as described previously (21). siRNA was transfected using Lipofectamine 2000 according to the manufacturer's instructions. The indicated plasmids were transfected using Superfect Reagent as described previously (20).

### Co-immunoprecipitation (Co-IP) and western blotting

Co-IP experiments were performed essentially as described previously (19,20). Briefly, for conventional IP experiments, HeLa cells were cultured in 10-cm culture dishes to 70% confluence. The cells were then either harvested without additional treatments or after treatment with either ionizing radiation (IR) or a specific genotoxic agent. The cells were then lysed with IP buffer (50 mM Tris HCl, pH 7.4, with 150 mM NaCl, 1 mM EDTA and 1% TRITON X-100) in the presence of a protease inhibitor mixture. In total, 1 mg of total proteins was used for each IP experiment. Additionally, the same Co-IP experimental procedure was used to obtain an IKKγ-enriched preparation in order to assess the relative abundance of IKKγ SUMOylation by western blot analysis using an anti-SUMO antibody. Similar results were obtained from two independent sets of experiments and the result of one of them is presented here. The gray values of the bands were calculated using the ImageJ software.

### Sequential two-step IP

HeLa cells were transfected with pcDNA3-FLAG or pcDNA3-FLAG-LRP16 using Superfect Transfection Reagent and stimulated with 50 μM VP16 for 30 min. Sequential immunoprecipitation was performed as follows: First, IP was performed with agarose beads conjugated with an anti-FLAG antibody (M2-agarose beads, Sigma). The proteins that were pulled down by the anti-FLAG antibody were then released by incubation with an M2-specific antigen, the 3×FLAG peptide (Sigma), according to the manufacturer's instructions. The released proteins that were obtained from the first round of IP were then used in a second round of IP with an anti-IKKγ antibody. The final products and inputs were then analyzed by western blot analysis. Reproducible results were obtained from two separate sets of experiments and the result of one of them is presented here.

### Luciferase assay

The cells (HeLa, C33A and MCF-7 cells) were seeded in 35-mm culture dishes and co-transfected with 0.5 μg of the 3×κB-luc reporter construct and the indicated siRNA or plasmid. pRL-SV40 (1 ng) was used as an internal control. The total amount of input DNA for each treatment was maintained constant by supplementing with pcDNA3.1. Forty-two hours after transfection, the cells were either left untreated or treated with 10 Gy IR or 50 μM VP16. The cells were then harvested for a luciferase assay using a Dual-Luciferase^®^ Reporter Assay System as described previously (19,20). The experiments were performed three times each with three sets of samples. The results of all three experiments were similar and one of them is presented.

### RT-PCR and real-time PCR

HeLa cells were transiently transfected with control or LRP16 siRNAs and treated with 10 Gy IR or 50 μM VP16 or left untreated. Four hours later, the total RNA was isolated with TRIzol reagent (Invitrogen). cDNA was synthesized using PrimeScript^TM^ reverse transcriptase (Takara Shuzo Co.) in accordance with the manufacturer's instructions. The RT-PCR samples were analyzed by agarose gel electrophoresis. Real-time PCR was performed using a SYBR Green PCR Reagent Kit (Takara Shuzo Co.), and the expression levels were calculated by the comparative Ct method (2^−ΔΔCt^) using β-actin expression as an internal control. All experiments were performed three times each with three sets of samples. The results for these experiments were all reproducible and therefore only one for each experiment is presented. The following primers were used: *LRP16* forward 5′-CCGCAGCGACATCACCAAGC-3′; *LRP16* reverse 5′-TCCGGCACTCGTCGGTAAGC-3′; *cIAP2* forward 5′-TTTTGCTGTGATGGTGGACTC-3′; *cIAP2* reverse 5′-TCTCCTGGGCTGTCTGATGTG-3′; *XIAP* forward 5′-CTTCCAAGAAATCCATCCA-3′; *XIAP* reverse 5′-TTCCAATCAGTTAGCCCTC-3′; *β-actin* forward 5′-AAAGACCTGTACGCCAACAC-3′; and *β-actin* reverse 5′-GTCATACTCCTGCTTGCTGAT-3′.

### Cell proliferation assay

The rate of cell proliferation was analyzed using a CCK-8 kit as described previously (24). Three biological replicates were prepared for each treatment and each assay was performed in triplicate.

### Immunofluorescence microscopy

The cells were immunostained as described previously (21). Fluorescence images were acquired with a confocal laser-scanning microscope (FV1000, Olympus).

### Detection of apoptosis

The frequencies of apoptosis were determined as described previously (21). Briefly, the cells were either untreated or subjected to the indicated treatments before being processed for apoptosis analysis. All experiments were performed three times, and for all experiments each sample was analyzed in triplicate. For each experiment, the result was quite reproducible and therefore only the result of one experiment is presented.

### Synthesis of PAR and auto-poly-ribosylation of PARP1

The auto-poly-ribosylation of PARP1 was synthesized with purified PARP1 as described previously (25). A 100-μl reaction mixture containing 3 μl of human PARP1 enzyme, 3 μl of 20 mM NAD or 250 μM Biotin-NAD, 10 μl of 10×activated DNA and 5 μl of 20×PARP buffer was incubated at 25°C for 1 h. The reaction mixture was then used directly in a PAR-binding assay or a GST pull-down assay.

### Recombinant protein expression and purification

For bacterial expression, IKKγ cDNA was cloned into pGEX-6P-1. The recombinant fusion protein expressing the pGEX-6p-1-LRP16 plasmid has been described in our previous report (19). The procedure used to obtain purified recombinant proteins from *E. coli* cells has been described previously (19). The pGEX-6p-1, pGEX-6p-1-LRP16 and pGEX-6p-1-IKKγ plasmids were transformed into BL21 *Escherichia coli* cells, and the cultures were induced at OD_600_ = 0.5∼1.0 with 100 mM IPTG for 10 h at 20°C. Crude bacterial lysates were prepared by sonication (nine rounds of 20-s bursts on ice) in lysis buffer (40 mM Tris-HCl, pH 7.5, 150 mM NaCl, 1 mM EDTA, 0.5% NP-40 and 10% (v/v) glycerol) in the presence of the protease inhibitor mixture. The GST-tagged recombinant proteins were then purified using the GST·Bind Resin according to the manufacturer's protocol.

### PAR binding assay

The recombinant proteins were incubated for 30 min at 32°C in 40 mM Tris-HCl, pH 7.5, 150 mM NaCl, 1 mM EDTA, 0.5% NP-40, 10% (v/v) glycerol and biotin-labeled PAR. The reaction mixtures were applied to nitrocellulose and washed for 30 min with TBS-T containing 100 mM NaCl. After incubation with streptavidin-HRP, the bound biotin-labeled PAR was detected using a DAB Horseradish Peroxidase Color Development Kit. Each sample was detected two times. Each experiment was performed two times and reproducible results were obtained.

### GST pull-down assay

The GST-tagged proteins that were associated with the GST·Bind Resin were incubated with various proteins of interest. The GST-pull down assay was performed as described previously (20). The total proteins that were associated with the beads were then analyzed by western blot analysis using the appropriate antibodies. Each sample of GST-pull down was analyzed two times. Each experiment was performed two times and reproducible results were obtained.

### Mass spectrometry

The bead-associated FLAG-tagged proteins of interest were eluted by incubation with the 3×FLAG peptide antigen (Sigma). The eluted products were fractionated on an SDS-PAGE gel. The gel was then subjected to silver staining treatment, and the visible bands were excised and submitted to mass spectrometry analysis.

### Design and synthesis of siRNA

The following siRNAs were ordered from GenePharma (Shanghai, China): LRP16-374, 5′-GCAGCGGGAGGAACATTAC-3′; LRP16-668, 5′-GACTGGCAAGGCCAAGATC-3′; PARP1-s1, 5′-AAGCCTCCGCTCCTGAACAAT-3′; PARP1-s2, 5′-AAGATAGAGCGTGAAGGCGAA-3′; siKu80, 5′-GACAGACACCCTTGAAGAC-3′; siKu70, 5′-GGATCATGCTGTTCACCAA-3′; and Scrambled, 5′-TTCTCCGAACGTGTCACGT-3′.

### Statistical analysis

The statistical analyses were performed using SPSS 13.0 for Windows (SPSS Inc, Chicago, IL, USA).

## RESULTS

### LRP16 physically interacts with nuclear IKKγ and PARP1

**Figure 1.**
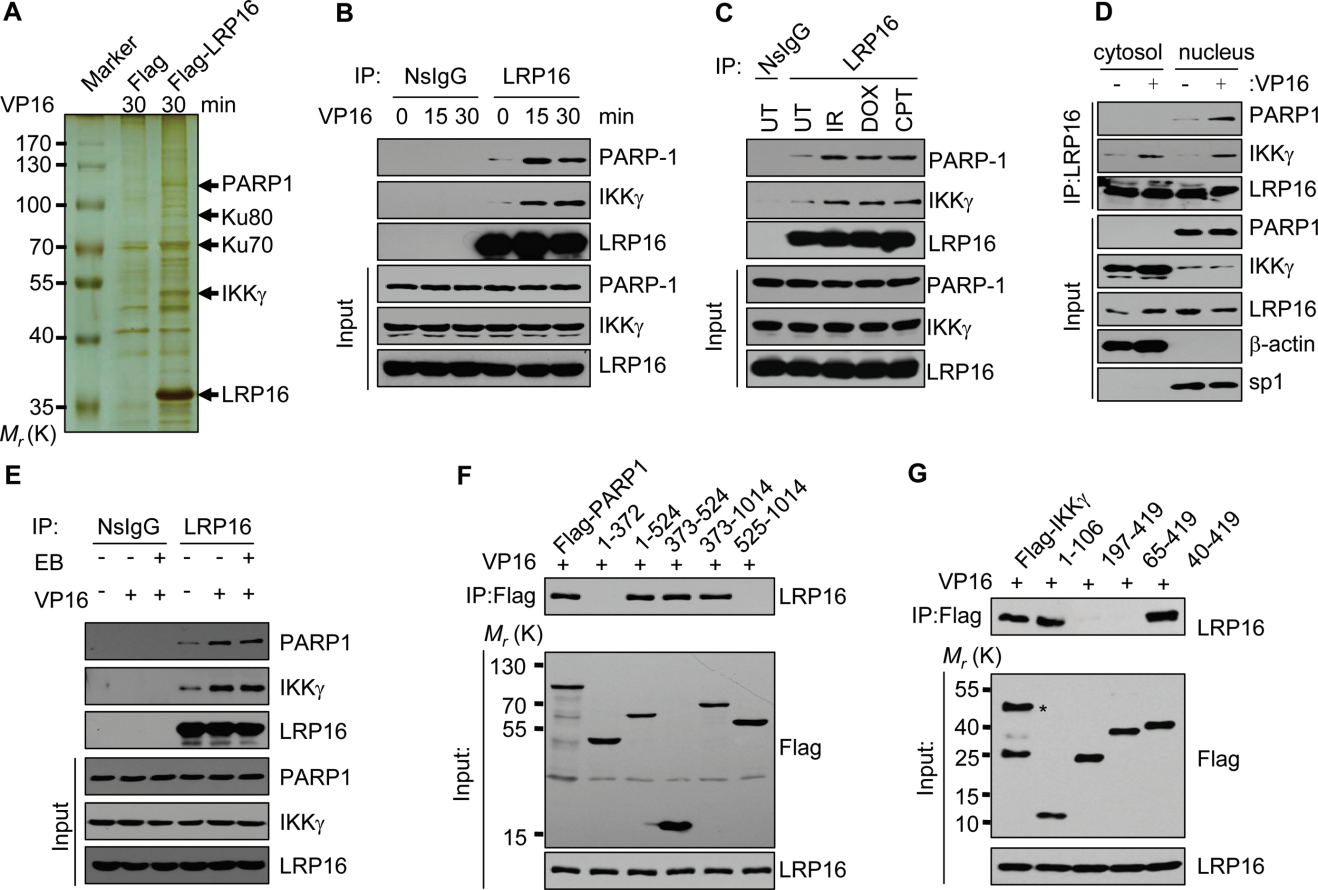
LRP16 physically interacts with IKKγ and PARP1. (**A**) A photograph of a representative silver-stained gel showing the profiles of proteins that were pulled down by IP. HeLa cells were treated with VP16 (50 μM) for 30 min. (**B**) HeLa cells were treated with 50 μM VP16 for the indicated times. Lysates were prepared for IP with an anti-LRP16 antibody or a rabbit IgG control. The IP products and 2% of the input were immunoblotted with the indicated antibodies. (**C**) Identical to B, except that total cell lysates from HeLa cells that were either exposed to IR (10 Gy) or treated with 5 μM doxorubicin (DOX) or 10μM camptothecin (CPT) for 30 min were used. (**D**) Identical to B, except that either the cytosolic or the nuclear fractions of the cell lysates that were derived from HeLa cells treated with or without 50 μM VP16 for 0 (-) or 30 (+) min were used. (**E**) Identical to B, except that ethidium bromide was added to a final concentration of 50 μg/ml when indicated. (**F**) HeLa cells were transfected with FLAG-tagged PARP1 deletion mutants and treated with VP16 for 30 min. The whole-cell extracts were prepared for Co-IP with an anti-FLAG antibody. (**G**) Identical to F, except that HeLa cells were transfected with FLAG-tagged IKKγ deletion mutants.

To explore the function of LRP16 in the DDR, we ectopically over-expressed FLAG-LRP16 in HeLa cells, treated the cells with the DSB-inducing agent VP16 (Supplementary Figure S1A), and then performed immunoprecipitation (IP) experiments using an anti-FLAG antibody to identify putative LRP16-interacting factors. To exclude nonspecific interactions, the FLAG-expressing vector was used as a control. The silver staining results showed that many new interaction partners are co-immunoprecipitated with the FLAG-agarose beads (Figure 1A), and the detailed results of the mass spectrometric analysis are provided in Supplementary Table S1. Interestingly, IKKγ and PARP1 were identified in the immunoprecipitates. Because modifications to nuclear IKKγ play a central role in the activation of the nuclear-to-cytoplasm NF-κB signaling pathway following DSB induction and because PARP1 constitutes a requisite for activating the DSB-induced NF-κB pathway by facilitating the concomitant ATM-mediated phosphorylation and PIASy-mediated SUMOylation of IKKγ (8,12,26,27), the mass spectrometry results prompted us to ask whether LRP16 plays any role in the regulation of this specific NF-κB pathway (referred to as the DSB-induced NF-κB pathway herein). Co-IP experiments were then performed, and the results showed that LRP16 is constitutively associated with PARP1 and IKKγ *in vivo* and that the interactions were significantly enhanced by VP16 and many other DSB-inducing agents, including IR, doxorubicin (DOX) and camptothecin (CPT) (Figure 1B, Supplementary Figure S1A, and Figure 1C). Alternatively, LRP16 was also effectively pulled down by anti-PARP1 or anti-IKKγ (Supplementary Figure S1B) from the same extracts. Furthermore, when nuclear or cytoplasmic extracts were used separately for the Co-IP using anti-LRP16 antibodies, both IKKγ and PARP1 were effectively pulled down from the nuclear extract, whereas only IKKγ was pulled down from the cytoplasmic extract at a reduced level compared with that obtained from the nuclear extract (Figure 1D). The addition of ethidium bromide for the disruption of DNA-protein interactions (28) in the IP experiments using anti-LRP16 antibodies did not have a major effect on the relative yield of the IKKγ pull-down (Figure 1E), indicating that such interactions, while enhanced by induction, do not require the physical presence of DNA. Similar results were also obtained when the extracts from MCF-7 and HT-29 cells were used (Supplementary Figure S1C and SD), demonstrating that these interactions are not a peculiar cell line-specific phenomena. Moreover, such interactions can be detected in whole-cell extracts derived from enucleated human red blood cells, but neither of these interactions was further enhanced by ionizing radiation (Supplementary Figure S1E), providing further support for the hypothesis that the effects observed in the extracts from cells with nuclei are indeed dependent on DSB induction rather than a co-incidental common effect due to the treatments with DSB-inducing agents or exposure to IR. Further experiments revealed that the deletion of the amino acid regions 1–524 or 373–1014, but not the amino acid regions 1–372 and/or 525–1014, greatly reduced the interactions with LRP16 (Figure 1F), identifying the region between amino acid 372 and 525 as a major determinant for the LRP16-PARP1 interaction. LRP16 could also be co-immunoprecipitated with FLAG-IKKγ(1–106) containing a helical domain (Figure 1G), defining the first 106 amino acids of IKKγ as sufficient for facilitating the interaction between LRP16 and IKKγ. Cumulatively, these data reveal that LRP16 is able to interact with PARP1 and IKKγ in various cell types and suggest that LRP16 is a key factor in the DNA damage-induced NF-κB signal transduction cascade.

### LRP16 is required for DSB-induced NF-κB activation

**Figure 2.**
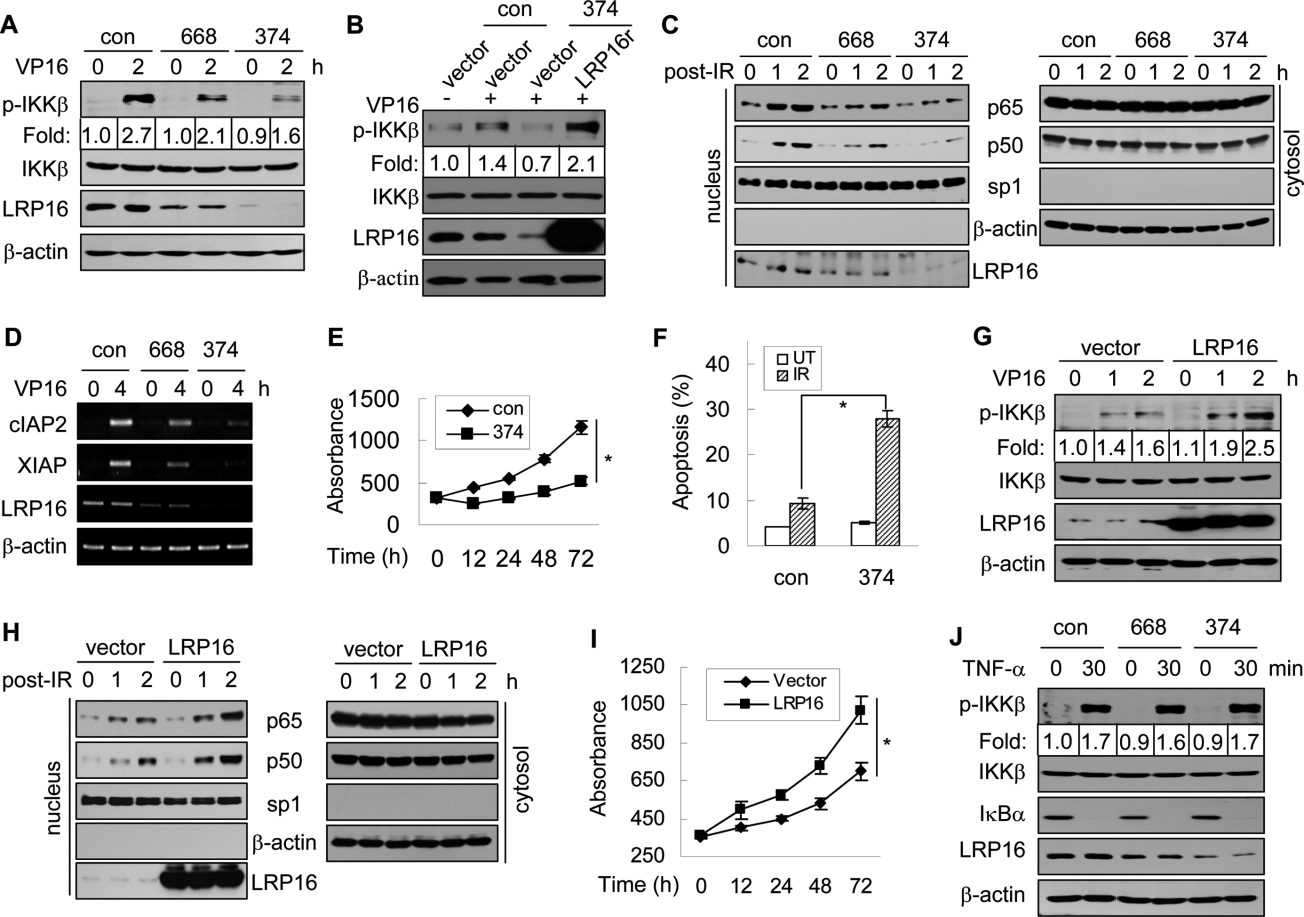
LRP16 is required for NF-κB activation in response to genotoxic stress. (**A**) HeLa cells were transfected with the indicated siRNA and treated with or without VP16. The lysates were used for immunoblotting with the indicated antibodies. (**B**) HeLa cells were transfected with the indicated plasmids and siRNAs. After 48 h, the cells were induced by 50 μM VP16 for the indicated times. The lysates were used for immunoblotting. (**C**) HeLa cells were transfected with the indicated siRNAs and exposed to 10 Gy IR. The cytoplasmic and nuclear fractions were then prepared and immunoblotted with the indicated antibodies. (**D**) The RNA expression levels of cIAP2 and XIAP were determined by RT-PCR. β-actin served as a loading control. (**E**) The absorbance values of individual cultures were measured at various time points (0, 12, 24, 48 and 72 h) after exposure to IR to assess the relative growth rate of the cultures. (**F**) The transfected HeLa cells were treated with 10 Gy IR. Twenty hours after treatment, the percentage of apoptotic cells was detected by Annexin V staining followed by fluorescence-activated cell sorting analysis. (**G**) C33A cells were transfected with the LRP16 expression vector or the control vector. Forty-eight hours after transfection, the cells were processed as in A. (**H**) Identical to C, except that C33A cells were used for the transfection with the indicated plasmids. (**I**) C33A cells were transfected with the indicated plasmids and then processed as in E 42 h after transfection. (**J**) HeLa cells were transfected with the indicated siRNAs and treated with 10 ng/ml TNF-α for the indicated times. The relative levels of phosphorylated IKKβ, total IKKβ, IκBα and LRP16 were determined. β-actin was used as a control. (E, F, I) Data represent the means ± SD (error bars) of three biological replicates analyzed in triplicate. *P<0.01.

The above-mentioned results prompted us to examine whether LRP16 might play a role in DNA damage-induced NF-κB activation. Two pairs of LRP16-siRNAs, namely siRNA-374 and -668, were used to deplete LRP16. We found that the depletion of LRP16 using either siRNA resulted in a pronounced reduction in the levels of the phosphorylated form but not in the overall level of IKKβ in HeLa cells, although a greater degree of reduction was found with siRNA-374 (Figure 2A, and Supplementary Figure S2A). The phenotype could be rescued by expressing a siRNA-resistant LRP16 vector (Figure 2B), demonstrating that the effect was indeed caused by LRP16 depletion. Moreover, this LRP16 depletion also resulted in a loss of NF-κB recruitment to the nucleus (Figure 2C), reduced levels of NF-κB-dependent luciferase reporter gene activity (Supplementary Figures S2B and S2C), impaired expression of NF-κB target genes (Figure 2D and Supplementary Figure S2D) and a reduced rate of cell proliferation (Figure 2E and Supplementary Figure S2E). The ectopic expression of IκBm, a dominant-negative mutant of IκBα that can blocks NF-κB activation (29), had an effect similar to that obtained with LRP16 depletion (Supplementary Figures S2F–S2H). Moreover, this LRP16 depletion also resulted in a significant increase in the frequency of apoptosis (Figure 2F and Supplementary Figures S2I and S2J). Conversely, the ectopic overexpression of LRP16 in C33A cells, in which the endogenous expression level of LRP16 is lower than that observed in other detected tumor cells (Supplementary Figure S2K), enhanced the phosphorylation of IKKβ (Figure 2G), the recruitment of NF-κB to the nucleus, the damage-dependent induction of the activity of NF-κB-dependent luciferase reporter gene and promoted cell growth/survival after DNA damage (Figure 2H and I and Supplementary Figure S2L–S2N). In contrast, the same LRP16 depletion did not have a major impact on TNFα-induced IKKβ phosphorylation, which, under our current experimental conditions, acted as an effective inducer for classical NF-κB activation but not DSBs (Figure 2J and Supplementary Figure S3). Thus, LRP16 is specifically required for the activation of the DSB-induced NF-κB signal transduction cascade but not for the activation of the classical NF-κB pathway (although LRP16 is apparently involved in the regulation of NF-κB-target gene expression once the classical NF-κB pathway is activated (21)).

### LRP16 is essential for IKKγ modifications and the PIASy-IKKγ interaction

**Figure 3.**
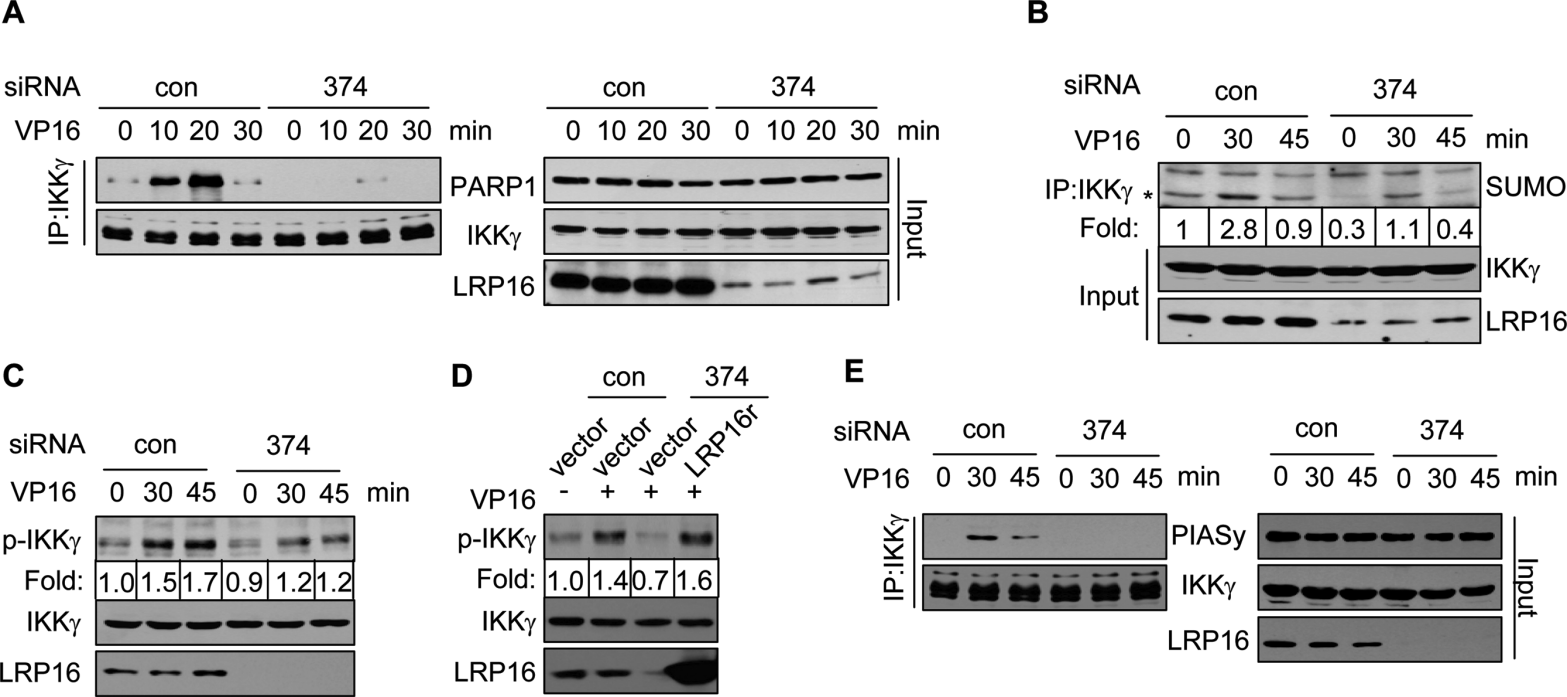
Depletion of LRP16 diminishes the PARP1-IKKγ interaction and IKKγ SUMOylation, and phosphorylation. (**A**) HeLa cells were first transfected with control scramble (con) or LRP16 siRNA 374 (374) and then treated with 50 μM VP16 for various times (0, 10, 20 and 30 min). Lysates were prepared from the treated cells and used to perform an IP experiment with an anti-IKKγ antibody. (**B**) HeLa cells were first treated with VP16 as in A. The total cell extracts were then used for IP with an anti-IKKγ antibody, and the IP products were analyzed by western blot analysis using an anti-SUMO1 antibody. (**C**) HeLa cells were treated as in B, and the relative amounts of phosphorylated IKKγ in individual samples were then assessed by western blot analysis using an anti-p-IKKγ antibody. (**D**) Identical to C, except that HeLa cells were co-transfected with the indicated siRNAs and plasmids. (**E**) HeLa cell lysates were prepared as in B, and the cell lysates were then used for the IP experiments. The relative levels of PIASy, IKKγ and LRP16 were then assessed by western blot analysis.

The dual physical interactions of LRP16 with both PARP1 and IKKγ within the nuclear compartment of human cells and the previous finding that the PARP-1-dependent assembly of IKKγ and PIASy is critical for IKKγ SUMOylation following DSB induction suggest that LRP16 may play an important role in DSB-induced IKKγ modifications (8,30). To investigate this possibility, we analyzed the DNA damage-induced IKKγ-PARP1 interaction and IKKγ modifications in LRP16-depleted HeLa cells. We found that LRP16 depletion resulted in a significant reduction of PARP1-IKKγ interactions (Figure 3A) and DSB-induced IKKγ modifications, namely SUMOylation and phosphorylation (Figure 3B and C). The transfection of cells in which LRP16 was depleted by siRNA 374 with an siRNA-resistant LRP16 vector diminished the effect of siRNA 374 on IKKγ phosphorylation (Figure 3D). In addition, we also found that LRP16 depletion led to a significant reduction in the PIASy-IKKγ interaction (Figure 3E).

### PAR binding is required for LRP16-PARP1 and LRP16-IKKγ interactions

**Figure 4.**
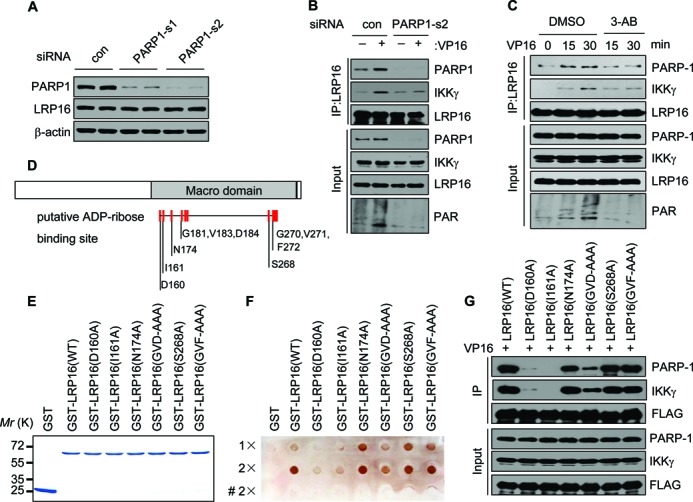
The LRP16-PARP1 and LRP16-IKKγ interactions are dependent on PAR. (**A**) HeLa cells were transfected with control or PARP1 siRNAs and the whole-cell extracts were examined with the indicated antibodies. (**B**) HeLa cells were transfected with the indicated siRNA and treated or not treated with VP16 (50 μM) for 30 min. IP was performed using an anti-LRP16 antibody. The samples were analyzed by immunoblotting with the indicated antibodies. (**C**) Identical to B except that lysates were derived from HeLa cells that were pretreated with either 10 mM 3-aminobenzamide (3-AB) or the solvent dimethyl sulfoxide (DMSO) for 0, 15 and 30 min were used. (**D**) A schematic illustration of LRP16 and its mutants. (**E**) GST, GST-LRP16 and GST-fusion LRP16 mutants were expressed using a prokaryotic expression system and the purified proteins were resolved by Coomassie staining. (**F**) PAR binding assays were performed with the indicated fusion proteins and synthesized PAR. ‘#’ represents the heat treatment. (**G**) HeLa cells were transfected with the indicated expression vectors of LRP16 mutants. Forty-eight hours after transfection, the cells were treated with VP16 and processed as in B, except that the antibody that was used for IP was anti-FLAG.

LRP16 binds both mono-ADP-ribose and PAR with high affinity (31), whereas PARP1-dependent PAR synthesis is linked to IKKγ modifications (8). Thus, the observation that LRP16 plays a critical role in PARP1-IKKγ interactions (Figure 3A) and DSB-induced IKKγ modifications prompted us to consider the possibility that the enhanced effect of DNA damage on LRP16-PARP1 and LRP16-IKKγ interactions is dependent on the binding of LRP16 to PAR. Indeed, we found that the depletion of PARP1 with two different PARP1-siRNAs in HeLa cells resulted in a marked reduction in the interaction between LRP16 and IKKγ (Figure 4A and B), whereas pretreatment with the PARP1 inhibitors 3-aminobenzamide (3-AB), benzamide (BEN), or PJ-34 all led to a marked reduction in the enhanced effect of DNA damage on the LRP16-PARP1 and LRP16-IKKγ interactions (Figure 4C and Supplementary Figure S4). Together, these data indicate that the unique affinity of LRP16 to PAR plays a critical role in facilitating its high-efficiency interactions with PARP1 and IKKγ following DSB induction.

LRP16 binds PAR, presumably through its macro domain (31). Indeed, we found that changing D160 and I161 of the macro domain of LRP16 to A160 and A161, was sufficient to significantly reduce its affinity to PAR, whereas the replacement of Gly 181, Val 183 and Asp 184 with Ala181, Ala183 and Ala184 only had a modest effect (Figure 4D–F and Supplementary Table S2). The replacement of D160 and I161 in LRP16 with A160 and A161 also greatly reduced its interaction with PARP1 and IKKγ (Figure 4G). Confocal analysis also showed considerable co-localization between ectopically expressed FLAG-LRP16 and PAR in HeLa cells treated with VP16, but less co-localization was obtained between ectopically expressed FLAG-LRP16 (I161A) and PAR (Supplementary Figure S5). Taken together, these data demonstrate that PAR plays a critical role in facilitating the enhanced interaction of LRP16 with both PARP1 and IKKγ and that the unique macro domain of LRP16 is required for facilitating this enhanced LRP16-PARP1 interaction following DSB induction.

### The O-Acetyl-ADP-ribose deacetylase and mono-ADP-ribosylhydrolase activity of LRP16 is not essential for DSB-induced NF-κB activation

Recent studies have described LRP16 as a novel deacetylase, that is able to deacetylate O-acetyl-ADP-riboses or to remove mono-ADP-riboses from ADP-ribosylated proteins (32–34). Thus, we wondered whether these activities may also be required for its role in DSB-induced NF-κB activation. Specifically, because it has been reported that Gly 270, Asn 174 and Asp 184 are required for both activities, we addressed this point by studying whether changing these residues would greatly affect the ability of LRP16 to interact with PARP1 and IKKγ. We found that the mutants LRP16 (G270A-V271A-F271A, or GVF-AAA) and LRP16 (N174A) still exhibited strong interactions with both PARP1 and IKKγ (Figure 4G). Similarly, LRP16 (Gly 181-Val 183-Asp 184, or GVD-AAA) could still interact with both PARP1 and IKKγ, even though the interaction with PARP1 was reduced compared with that of wild-type LRP16 (Figure 4G). Additionally, the ectopic expression of LRP16 (N174A) and LRP16 (GVF-AAA) potently up-regulated NF-κB reporter gene activity (Supplementary Figure S6). Thus, neither the O-Acetyl-ADP-ribose deacetylase nor the mono-ADP-ribosylhydrolase activity of LRP16 is required for its role in mediating DSB-induced NF-κB activation.

### LRP16, IKKγ and PARP1 constitutively form complexes

**Figure 5.**
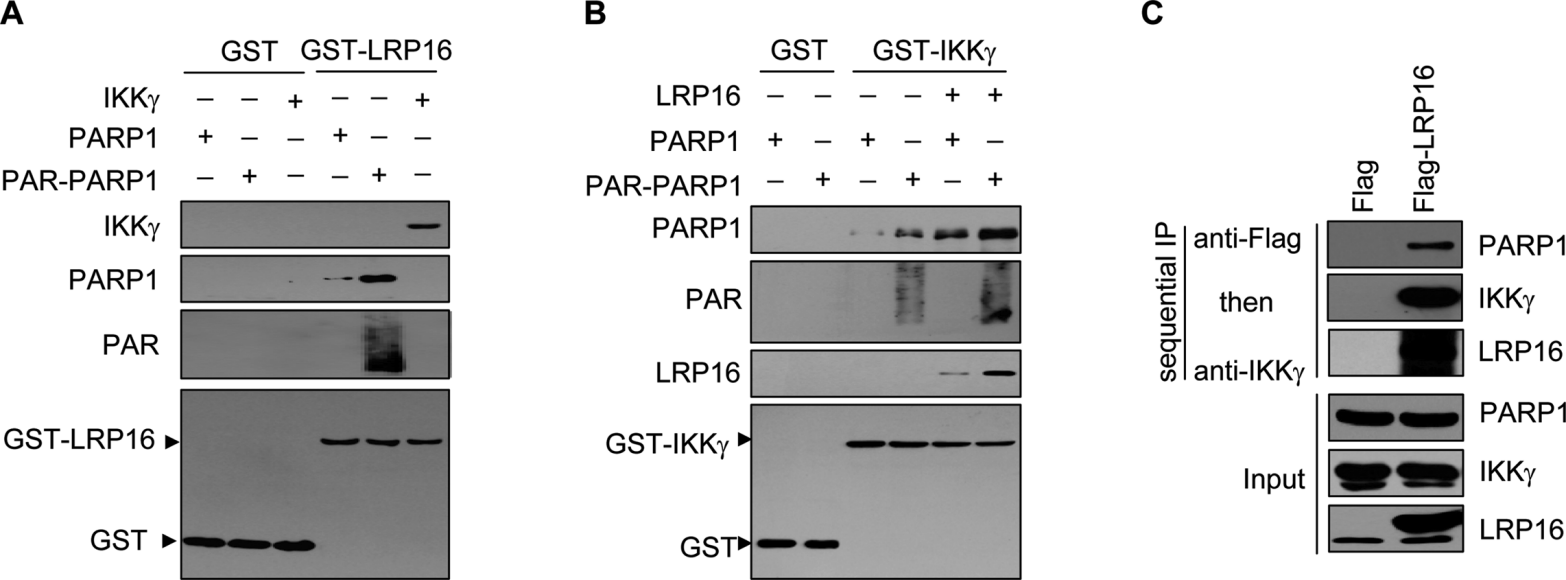
LRP16, IKKγ and PARP1 could reside in the same complexes. (**A**) Various combinations of purified IKKγ, commercial PARP1 and GST-LRP16 (or GST) were mixed and incubated under in vitro conditions for 2 h at room temperature. A GST pull-down assay was then performed with GST-LRP16. (**B**) Identical to A, except that IKKγ and GST-LRP16 were replaced with GST-IKKγ and LRP16, respectively, to study the binding partners of IKKγ. (**C**) HeLa cells were transfected with either FLAG or FLAG-LRP16 expression vectors for 48 h and then treated with 50 mM VP16 for 30 min. Total cell lysates were then prepared for sequential IP experiments (with an anti-FLAG antibody followed by an anti-IKKγ antibody).

It has been well established that high levels of the PARP1-IKKγ interaction are detected only after DNA damage (12). Remarkably, however, we noted that the LRP16-PARP1 and LRP16-IKKγ interactions are readily detectable (Figure 1B–E) even in the absence of DNA damage induction. Thus, we reasoned that because of its ability to interact with both PARP1 and IKKγ, LRP16 might serve as a mediator to facilitate the rapid recruitment of both PARP1 and IKKγ onto individual DSB damage sites to promote the concomitant SUMOylation and phosphorylation of IKKγ by recruiting both ATM and PIASy onto individual IKKγ molecules. Indeed, we found that recombinant GST-LRP16 could interact with both PARP1 and IKKγ under *in vitro* conditions (Figure 5A). Moreover, although GST-IKKγ can interact with both PARP1 and PAR-PARP1, the interactions were significantly enhanced by LRP16 (Figure 5B). Thus, under *in vitro* conditions, LRP16 could not only directly interact with both PARP1 and IKKγ but also enhance the interaction between PARP1 and IKKγ. Significantly, a two-step IP experiment using an anti-FLAG antibody (specific for FLAG-LRP16) followed by an anti-IKKγ antibody in FLAG-LRP16-expressing cells resulted in the pull-down of not only FLAG-LRP16 and IKKγ but also PARP1 (Figure 5C). These data, therefore, provide a piece of evidence for the existence of a type or types of complexes that contain LRP16, PARP1 and IKKγ.

### The role of LRP16 in mediating DSB-induced NF-κB activation is dependent on Ku70/Ku80

**Figure 6.**
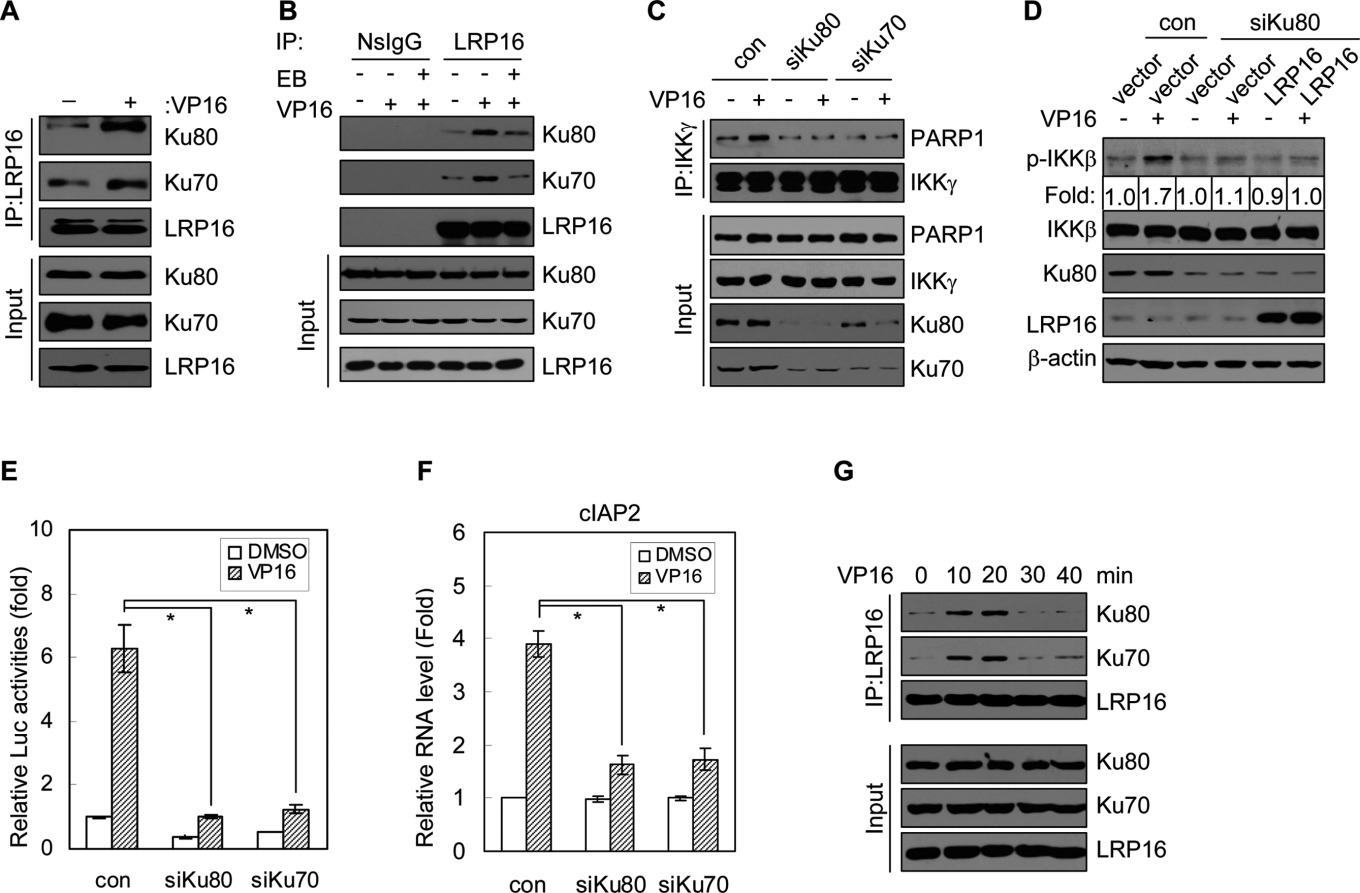
LRP16 indirectly interacts with both Ku70 and Ku80. (**A**) Results of western blot experiments showing the physical interaction between endogenous LRP16 and Ku70/Ku80 in HeLa cells with (+) and without (-) VP16 treatment (50 μM). (**B**) Identical to A, except that ethidium bromide (50 μg/ml) was included in the lysates of the corresponding wells to test for DNA-mediated protein interactions. (**C**) Effects of siRNA-mediated depletion of Ku80 or Ku70 on the interaction of IKKγ with PARP1. (**D**) HeLa cells were co-transfected with the indicated plasmids and siRNA, and the lysates were immunoblotted with the indicated antibodies. (**E** and **F**) Effects of siRNA-mediated knockdown of Ku80 or Ku70 on DNA damage-induced NF-κB reporter gene activity (E) and the mRNA level of cIAP2 (F). Data represent the means ± SD (error bars) of three biological replicates analyzed in triplicate. *P<0.01. (**G**) Results of western blot experiments showing the dynamic interaction between endogenous LRP16 and Ku70 or Ku80 in HeLa cells that were treated with VP16 (50 μM).

The existence of complexes that contain both PARP1 and IKKγ can promote the rapid recruitment of these two essential factors to DNA damage sites and, therefore, increase the chance of the subsequent PAR-dependent recruitment of both ATM and PIASy and the concomitant modifications of IKKγ by ATM and PIASy. This intriguing prospect prompted us to ask whether other component(s) may also be part of these complexes. We noted that Ku70 and Ku80 were also among this short list of putative LRP16-interacting factors (Figure 1A and Supplementary Table S1). The notion that LRP16 also interacts with both Ku70 and Ku80 is interesting because the Ku70/Ku80 complex binds specifically to DSBs and that DSBs are a specific type of lesion that can activate the DNA damage-induced NF-κB pathway (35–39). Follow-up experiments confirmed that LRP16 indeed interacts with both Ku70 and Ku80 in HeLa cells (Figure 6A). To rule out the possibility that these interactions are indirect and mediated by DNA/chromatin, ethidium bromide was added to the lysates for IP. Unexpectedly, ethidium bromide was able to markedly impair the VP16 induced-interaction between LRP16 and Ku70/Ku80, which indicates that these interactions are DNA-dependent (Figure 6B). Because Ku70 and Ku80 are among the first factors to be recruited to DSB damage sites, we wondered whether Ku70 and Ku80 might also serve as mediators for promoting the rapid and specific recruitment of LRP16 and/or PARP1 and IKKγ onto DSB damage sites. We found that the siRNA-mediated depletion of Ku70 or Ku80 resulted in significant attenuation of the PARP1-IKKγ interaction (Figure 6C), and that the depletion of Ku80 is able to inhibit the IKKβ phosphorylation enhanced by LRP16 in the presence of DNA damage (Figure 6D). Moreover, the knockdown of either endogenous Ku80 or Ku70 by siRNA markedly diminished the magnitudes of NF-κB reporter gene activity and NF-κB target gene expression following DSB induction (Figures 6E and 6F). In addition, we found that LRP16, PARP1 and Ku80 exhibit similar patterns of distribution within the nuclei, whereas IKKγ was detected in both the nuclei and the cytoplasm (Supplementary Figure S7). Thus, it appears that LRP16, through its physical interactions, plays a critical role in the efficient and targeted assembly of IKKγ and PARP1 at DSB damage sites to ensure the DSB-specific activation of NF-κB. Intriguingly, however, Ku70 and Ku80 are also required for the repair of DSBs. Therefore, Ku70 and Ku80, which are involved in two distinct functions, namely NF-κB activation and DSBs repair, respectively, must fulfill these two different functions either as two separate populations or within the same entity but in a sequential manner. Interestingly, we found that although the interactions among LRP16, Ku70 and Ku80 were enhanced shortly after DSB induction, these interactions were rapidly diminished thereafter (Figure 6G). Thus, it appears that the LRP16-Ku70/Ku80 interactions are transient in nature, and this finding supports the second model, i.e. the Ku70/Ku80 complex functions in the following sequential manner: first to facilitate the activation of the DSB-induced NF-κB signal transduction cascade through its interaction with LRP16, and then ‘free’ itself from LRP16 to participate in the DSB repair process.

## DISCUSSION

**Figure 7.**
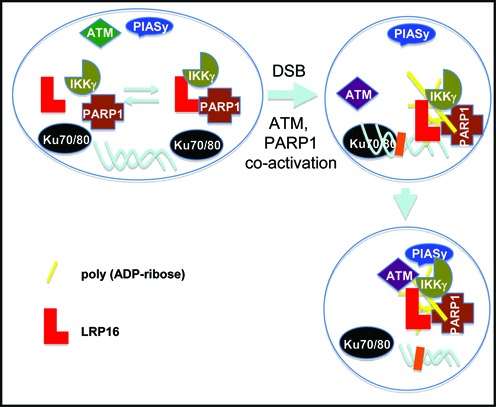
A schematic model illustrating the proposed interactions among the key components that are involved in the DSB-induced NF-κB activation. Briefly, weak constitutive interactions exist among LRP16, PARP1, IKKγ, Ku70 and Ku80 in unperturbed human cells. Following DSB damage induction, LRP16, PARP1 and IKKγ are recruited to DSB damage sites via their indirect association with Ku70 and Ku80. In contrast, DSB also cause the activation of ATM and the auto-poly-ribosylation of PARP1. The newly formed PAR-PARP1 then serves as a platform for recruiting the PAR-binding protein ATM and PIASy into the complex, which can ultimately result in the simultaneous phosphorylation and SUMOylation of IKKγ and the activation of NF-κB. In the absence of DSB damage, the activation of ATM or PARP1 and, accordingly, the phosphorylation or SUMOylation of IKKγ may occur. However, the simultaneous occurrence of these two modes of phosphorylation and SUMOylation of IKKγ modifications and, therefore, NF-κB activation either do not occur or occur only at low frequencies.

The concomitant phosphorylation and SUMOylation of nuclear IKKγ is the key requisite for activating the DSB-induced NF-κB pathway (26,30,40,41). Interestingly, DSBs serve as an initial common cue for the activation of both ATM and PARP1 (42–45), whereas the subsequent damage-induced poly(ADP-ribosyl)ation of PARP1 establishes PAR-PARP1 as a scaffold for recruiting both ATM and PIASy onto IKKγ. ATM and PIASy could interact with the scaffold through their PAR-binding domains, thereby promoting the concomitant phosphorylation and SUMOylation of individual nuclear IKKγ molecules (8,46). However, although genotoxic cues other than DSBs could also result in the activation of both ATM and PARP1, and similarly, IKKγ phosphorylation and SUMOylation, they do not necessarily cause the activation of NF-κB (12,40,47). Furthermore, it has been recognized that DNA damage-induced PAR-PARP1 is short-lived in nature, whereas activated ATM can be relatively stable (48–51). Thus, the successful recruitment of both ATM and PIASy onto individual short-lived PAR-PARP1 scaffolds must occur not only in a lesion (DSBs)-specific manner but also in a highly efficient and coordinated fashion shortly after damage induction. In this study, we show that LRP16, which is a PAR-binding protein, constitutively interacts with PARP1 and IKKγ and indirectly interacts with the Ku70/Ku80 DSB-binding complex. Additionally, LRP16 is required for the establishment of strong interactions among PARP1, IKKγ and PIASy and, accordingly, the phosphorylation and SUMOylation of IKKγ in response to DSB (Figure 7). Taken together, our data suggest that LRP16 functions not only to provide the lesion-specificity of the response, because of its unique interactions with both the DSB-specific Ku70 and Ku80, but also to ensure the successful PAR-PARP1-dependent recruitment of both ATM and PIASy along with IKKγ. These highly coordinated protein-protein interactions, therefore, ensure the efficient formation of the NF-κB activation signalosome in a DSB-specific manner despite the short-lived nature of the damage-induced PAR-PARP1. Therefore, our studies have provided important new mechanistic insights regarding the regulation of the DSB-specific NF-κB pathway and identified new potential targets for manipulating this important signaling cascade.

The fate of mammalian cells that have experienced damage through DSBs is an important research topic for several reasons. First, the survival of some of these cells that have experienced programmed DSBs, i.e. lymphocytes that have undergone V(D)J recombination and germs cells that have completed homologous recombination, is an important requisite for the generation of genetic diversities that are dependent on recombination-mediated DSB repair (52–54). In addition, the elimination of those cells that have encountered incidental DSBs but have failed to repair the DSBs in an appropriate manner is critical for preventing the accumulation of mutations and suppressing oncogenesis. However, in the context of certain clinical settings, such as in some anti-cancer treatments that involve cytotoxic IR or DSB-inducing anticancer drugs, maximizing the killing effect of DSBs toward cancer cells may be a key determinant for increasing the therapeutic benefits of the treatments. In this regard, the discovery of NF-κB activation, the key step in a pro-survival and pro-inflammatory pathway, as a new dimension of DDR has opened new opportunities for understanding and addressing these important issues. Therefore, the new insights that have been discovered by our study will prove significant in this endeavor. In particular, the identification of LRP16 as an essential new factor in this specific pathway will offer unique opportunities for determining biological roles, such as roles in lymphocyte development and immunity, and/or for pharmacologically manipulating the functional output of this pathway for therapeutic gain. In particular, future experiments, including knockout studies of LRP16 in mice or other model organisms, should investigate this important aspect of the DDR.

## SUPPLEMENTARY DATA

Supplementary Data are available at NAR Online.

SUPPLEMENTARY DATA
