# Supplementary material for: An LRP16-containing preassembly complex contributes to NF-κB activation induced by DNA double-strand breaks
Source: Nucleic Acids Res. 2015 Mar 3;43(6):3167–79. doi: 10.1093/nar/gkv161 (PMC4381070; doi:10.1093/nar/gkv161)
Supplement: SUPPLEMENTARY DATA [file supp_43_6_3167__index.html]

An LRP16-containing preassembly complex contributes to NF-κB activation induced by DNA double-strand breaks — SUPPLEMENTARY DATA 

# An LRP16-containing preassembly complex contributes to NF-κB activation induced by DNA double-strand breaks

## SUPPLEMENTARY DATA

**Files in this Data Supplement:**

- Supplemental Tables and Figures
