# Supplementary material for: An LRP16-containing preassembly complex contributes to NF-κB activation induced by DNA double-strand breaks
Source: Nucleic Acids Res. 2015 Mar 3;43(6):3167–79. doi: 10.1093/nar/gkv161 (PMC4381070; doi:10.1093/nar/gkv161)
Supplement: SUPPLEMENTARY DATA [file supp_gkv161_nar-02806-d-2014-File009.doc]

**An LRP16**-**Containing Preassembly Complex Contributes to NF**-**κB Activation** **Induced** **by DNA Double Strand Breaks**

Zhiqiang Wu, Chunmeng Wang, Miaomiao Bai, Xiaolei Li, Qian Mei, Xiang Li, Yao Wang, Xiaobing Fu, Guangbin Luo, Weidong Han

Online supplemental information

Supplemental figures: 7

Supplemental tables: 2

**Figure S1. LRP16 physically interacts with PARP1 and IKKγ in several human cell lines.** (A) HeLa cells were stimulated with 50 μM VP16 for 30 min or exposed to 10 Gy ionizing radiation and stained with anti-γ-H2AX followed by Alexa-Fluor 594 goat anti-mouse IgG. The nucleus was counterstained with DAPI. Bar = 20 μm. (B) Lysates from HeLa cells without an exogenous stimulus were used for IP with anti-PARP1 or anti-IKKγ, and the IP products and the corresponding inputs were analyzed by western blot analysis. Individual western blots with different antibodies were performed separately. (C, D) Identical to B, except that IPs were performed with cell lysates that were derived from either untreated (-) or VP16-treated **(+)** MCF-7 (C) and HT-29 (D) cells with an anti-LRP16 antibody. (E) Identical to B, except that IPs were performed with cell lysates from either untreated (-) or 10 Gy IR-exposed **(+)** red blood cells.

**Figure S2. LRP16 is essential for NF-κB activation in response to genotoxic stress.** (A) HeLa cells were transfected with control or LRP16-siRNAs and then exposed to 0 or 10 Gy ionizing radiation (IR), and cell lysates from these cells were then prepared for western blot experiments. (B, C) The relative levels of expression of an NF-κB reporter target gene in wild-type HeLa cells (or MCF-7 cells) or in their LRP16-depleted counterparts, after treatment with or without 10 Gy IR (or 50 μM VP16), were assessed. Briefly, the cells were co-transfected with 3×κB-luc and LRP16 siRNA or control siRNA. Forty-two hours after transfection, the cells were treated with IR or VP16, and luciferase assays were performed 7 h later. (D) Real-time PCR was performed to quantify the RNA expression levels of cIAP2 and XIAP in HeLa cells that were treated with IR (10 Gy) or VP16 (50 μM). (E) HeLa cells transfected with control or LRP16 siRNAs (668 and 374) and exposed to various doses of IR. The cell proliferation rate was quantified with CCK-8. (F, G, H) NF-κB-dependent luciferase reporter gene activity, mRNA expression of cIAP2, and cell proliferation were detected in IκBm and LRP16 siRNA transfected HeLa cells treated with or without radiation (10 Gy). (I, J) Representative results of apoptosis in HeLa and MCF-7 cells are shown. Cells that were transfected with control or LRP16 siRNAs were left treated or untreated with VP16 (100 μM) and IR (20 Gy). (K) Whole-cell extracts were prepared for western blot analysis with anti-LRP16 and anti-β-actin antibodies. (L, M) C33A cells were co-transfected with 3×κB-luc and the LRP16 expression vector. Forty-two hours after transfection, the cells were processed as in B. (N) C33A cells were transfected with the LRP16 expression vector or the empty vector and processed as in E. (B-H, L-N) The data are presented as the means ± SD (error bars) of three biological replicates analyzed in triplicate. **P<0.01, ^#^P >0.05.*

**Figure S3. TNF-α induced DNA damage.** HeLa cells were treated with TNF-α (20 ng/ml) for the indicated amount of time and then assayed by IF. Scale bar, 20 μm.

**Figure S4. Effect of PARP1 inhibition on the LRP16-PARP1 and LRP16-IKKγ interactions in HeLa cells.** Treatment with PARP1 inhibitors resulted in reduced LRP16-PARP1 and LRP16-IKKγ interactions in HeLa cells following exposure to 10 Gy ionizing radiation.

**Figure S5. Analysis of PAR binding *in vitro*.** Confocal microscopy of FLAG-tagged LRP16 (or mutant) (red) and PAR (green) in HeLa cells overexpressing FLAG-LRP16 or FLAG-LRP16 (I161A) was performed after stimulation for 15 min with VP16. Scale bar = 10 µm.

**Figure S6. Effects of LRP16 mutants on DNA damage-induced NF-κB reporter gene activity.** The relative levels of luciferase activity were normalized to the activity of the empty expression vector group, which was arbitrarily set to a value of 1. All of the experiments encompassed three independent biological replicates, and each sample was analyzed in triplicate. The results are expressed as the means ± SD (error bars). **P<0.01*.

**Figure S7. Co-localization of Ku80 with LRP16, PARP1 and IKKγ.** HeLa cells were stimulated with 50 μM VP16 for 15 min and stained with rabbit anti-LRP16, rabbit anti-PARP1, rabbit anti-IKKγ and mouse anti-Ku80 primary antibodies and then with Alexa-Fluor 488 goat anti-rabbit IgG and Alexa-Fluor 594 goat anti-mouse IgG. The nucleus was counterstained with DAPI. Scale bar, 20 μm. The images were captured using a confocal microscope.

**Table S1. D**etailed results of the mass spectrometric analysis.

| Protein name | Locus | Description | Functions |
| --- | --- | --- | --- |
| Ku70  Ku80 | NP_001460.1  NP_066964.1 | Regulatory subunit of DNA-PK  Regulatory subunit of DNA-PK | DNA repair |
| PARP1  IKK gamma  IKK beta  ERC1  PKR | NP_001609.2  NP_003630.1  NP_001547.1  NP_829884.1  NP_002750.1 | Poly(ADP-ribosyl)transferase  Regulatory subunit of the canonical IKK complex  Component of the canonical IKK complex  Regulatory subunit of the IKK complex  Double stranded RNA activated protein kinase | NF-kappaB activation |
| HNRNPU  CSTF3  YBX1  DHX9  DDX21 | NP_114032.2  NP_001317.1  NP_004550.2  NP_001348.2  NP_004719.2 | Belongs to the subfamily of hnRNPs  Required for polyadenylation and 3'-end cleavage of mammalian pre-mRNAs  Nuclease sensitive element binding protein  Unwinds double-stranded DNA and RNA in a 3' to 5' direction  Unwinds double-stranded RNA | pre-mRNA processing |
| PPP2R1A | NP_055040.2 | Protein phosphatase 2 | Chromosome segregation |
| LRP16 | NP_054786.2 | Bait |  |

**Table S2. Binding activities of the LRP16 mutants.**

| protein | Mutational site(s) | Poly(ADP-ribose) binding |
| --- | --- | --- |
| LRP16 wild type |  | ++++ |
| D160A | 160 | + |
| I161A | 161 | ± |
| N174A | 174 | ++++ |
| GVD-AAA | 181, 183, 184 | ++ |
| S268A | 268 | ++++ |
| GVF-AAA | 270, 271, 272 | ++++ |
